# Morphological, microbiological and ultrastructural aspects of sepsis by *Aeromonas hydrophila* in *Piaractus mesopotamicus*

**DOI:** 10.1371/journal.pone.0222626

**Published:** 2019-09-20

**Authors:** Fausto A. Marinho-Neto, Gustavo S. Claudiano, Jefferson Yunis-Aguinaga, Victor A. Cueva-Quiroz, Karina K. Kobashigawa, Nathan R. N. Cruz, Flávio R. Moraes, Julieta R. E. Moraes

**Affiliations:** 1 Departament of Veterinary Pathology, School of Agricultural and Veterinary Sciences, São Paulo State University (UNESP), Jaboticabal, São Paulo, Brazil; 2 Institute of Biodiversity and Forests, Federal University of Western Pará (UFOPA), Santarém, Pará, Brazil; 3 Aquaculture Center of UNESP, Jaboticabal, São Paulo, Brazil; 4 Departament of Veterinary Surgery and Clinic, School of Agricultural and Veterinary Sciences, São Paulo State University (UNESP), Jaboticabal, São Paulo, Brazil; Universitat de Barcelona, SPAIN

## Abstract

*Aeromonas* bacteria can cause an infection characterized by septicemia and is one of the most common pathogens in tropical fish. This disease is responsible for high morbidity and mortality rates, causing considerable losses in aquaculture. Thus, the understanding of its pathophysiology is crucial to develop control strategies of this bacterial infection in farmed fish. This study aimed to characterize early pathological aspects of acute sepsis in pacu (*Piaractus mesopotamicus*) experimentally infected with *Aeromonas hydrophila*. A total of 160 juvenile pacus were inoculated intraperitoneally with *A*. *hydrophila* (1.78 x 10^9^ CFU/mL) and at 0 (control), 1, 3, 6, and 9 hours post-inoculation (hpi), animals were anesthetized and samples were collected for microbiological, light microscopy and transmission electron microscopy (TEM) analyzes. The results showed the occurrence of hemodynamic alterations, such as hemorrhage and congestion, which were observed mainly after 6 and 9 hpi. It was possible to re-isolate *Aeromonas* at all sampling times except in control group. However, just after 9 hpi it was possible to find the bacteria in all fish and tissues. Light microscopy analyses revealed a degenerative process, necrosis and vascular damage mainly at 6 and 9 hpi. According to the ultrastructural examination, areas of cellular death were identified in all examined tissues, especially at 6 and 9 hpi. However, the most severe, related to necrosis, were observed after 6 and 9 hpi. The findings suggested that this bacterium spreads in the first hpi through the fish organs, mainly affecting spleen, liver and kidney, causing irreversible lesions at the molecular level.

## Introduction

Over the past five decades, world fish production has grown twice as fast as human population and the consumption *per capita* increased from 9.9 kg in 1960 to 20.1 kg in 2014 [1]. In Brazil, pacu (*Piaractus mesopotamicus*) is one of the most common reared freshwater fish species [2]. Both growth and intensification of fish production has increased the incidence and severity of diseases, especially those of bacterial origin, such as aeromonosis caused by *Aeromonas* spp., which has a great significance in intensive rearing systems, due to its high mortality rates [3].

Among motile *Aeromonas* species, strains of *A*. *hydrophila* are considered the most virulent for teleosts [4,5] and it is also a zoonosis that causes diarrhea and septicemia in humans [6,7]. In fish, the infection by *Aeromonas* spp. causes rupture of small blood vessels, leading to cutaneous and fins hemorrhage, progressing to ulcerations with loss of epithelium, anemia, anorexia, lethargy, hemorrhagic sepsis and death [8,9].

The deleterious effects caused by Gram-negative bacteria, such as *A*. *hydrophila*, are consequences of endotoxins, triggering sepsis or endotoxemia; complex syndromes that are defined by the presence of a systemic inflammatory response (SIRS) [10,11]. The consequence of this exacerbated and uncontrolled inflammatory response is the appearance of the lesions in multiple organs and high mortality [12]. Internal hemorrhagic injuries associated to high mortality are common manifestations in acute and super-acute aeromonosis caused by *A*. *hydrophila* in fish [13]. Similarly, Claudiano et al. [11] observed the firsts signs of *Aeromonas* infection, wich were petechiaes and suffusions, 9 hours post-inoculation of *A*. *hydrophila* in pacus, showing the importance of understanding aeromonosis.

Based on the aforementioned, this study aimed to characterize by morphological, ultrastructural and microbiological studies, the early stages of the septic process in different tissues of pacus experimentally infected with *A*. *hydrophila*.

## Material and methods

### Fish and maintenance conditions

A total of 50 healthy pacus (*P*. *mesopotamicus*) (250 ± 99.3 g and 15 ± 2 cm), originating from the same spawning of fish rared at Fish Repoduction Laboratory of Aquaculture Center of Unesp (Caunesp), were randomly distributed in five fiber tanks of 250 L (*n* = 10 fish/tank), supplied with chlorine-free running water from spring, at a flow of one liter per minute and constant aeration. Fish were acclimated for two weeks before the experiment. During this period, fish were fed to apparent satiety twice a day. Tanks were siphoned once a week and water quality was monitored daily using multiparameters probes (YSI® model 55 and model 63—YSI, Yellow Springs, OH, USA). Water quality parameters were kept as follows: dissolved oxygen = 5.1 ± 0.6 mg.L^-1^; temperature = 29.47 ± 1.58°C; pH = 7.66 ± 0.36 and electrical conductivity = 117.96 ± 6.12 μScm^-1^. Parameters remaining within the adequate ranges for this species [14,15]. Ethical protocol for this study was approved by Ethics committee (CEUA-UNESP) under protocol number (01471/15) in accordance with guidelines for care and use of laboratory animals of Sao Paulo State University, Jaboticabal, Brazil.

### Bacterial strain and preparation of challenge suspension

*Aeromonas hydrophila* strain isolated from skin lesion of naturally infected pacus during an outbreak was used in this study. For genetic characterization, the bacterial mass originated from the culture of pure colonies underwent a DNA extraction process, according to the manufacturer’s methodology (“Genomic DNA Purification Kit—Wizard®”). DNA concentration was 1690.9 ng/μL and absorbance ratio 260/280 and 260/230, varying between 2.02 and 2.04. Subsequently obtaining the DNA, ribosomal gene 16S rRNA was amplified according to Sarcar et al. [16]. Sequences were analyzed by the BLAST algorithm (http://www.ncbi.nlm.nih.gov), which presented 99% similarly with *A*. *hydrophila* (Accession number: CP007518-2). The challenge strain was grown on tryptic soy agar (TSA) (Kasvi, São José do Pinhais, PR, Brazil) and incubated at 28°C for 24 h. Bacterial suspensions were prepared by transferring a single colony of *A*. *hydrophila* to Falcon tubes with 50 mL of tryptic soy broth (TSB) (Kasvi, Brazil) and reincubated at 28°C in a bacteriological incubator (New Brunswick Scientific, New Jersey, NJ, USA). Twenty-four hours after incubation bacterial suspensions were centrifuged (Sorvall Legend Mach 1.6R, Germany) at 1792 *g*/10 min/4°C. Bacterial mass was resuspended in sterile 0.65% NaCl solution. Bacterial concentration was estimated by spectrophotometry and confirmed by determining the colony-forming units (CFU) of the original culture (serial dilutions, plating, and colony counting), reaching a concentration of 1.78 x 10^9^ CFU/mL.

### Experimental design and sepsis induction

After acclimation period, 50 pacus were randomly distributed into five groups for biological samples obtainment, according to time in hours post-inoculation (hpi): control group or 0 hpi; group 1 hpi; group 3 hpi; group 6 hpi and group 9 hpi (*n* = 10). Control group fish were injected by intraperitonal route with 0.5 mL of sterile 0.65% NaCl solution, while fish from the other groups were inoculated by the same route with 0.5 mL of *A*. *hydrophila* solution (1.78 x 10^9^ CFU/mL) [11]. Specimens were maintained in fasting during the 24 h previous challenge. Before the procedures, all fish were anesthetized by immersion in benzocaine solution (0.1 gL^-1^) [17].

### Sampling of biological material

After 0, 1, 3, 6 and 9 hpi, fish were anesthetized for blood sampling collection by puncture of the caudal vein using with sterile needles and syringes. Blood was immediately used for microbiological examination. After blood collection, anesthetized fish were euthanized by pithing and submitted to necropsy. Fragments of the spleen, heart, brain, liver and anterior kidney were aseptically sampled after the procedures of antisepsis and necropsy using sterile surgical instruments (e.g., scissors, tweezers, blades), aiming to perform microbiological, histopathological and ultrastructural examinations. Additionally, intestine, gills and pancreas were sampled for the histopathological exam.

### Clinical trial and macroscopic alterations

Animals were evaluated continually throughout the experiment, to verify possible macroscopic alterations and/or clinical behavior.

### Microbiological analyzes

In all sampling times, an aseptically removed fragment of each sampled organ and fresh blood were immediately streaked with a platinum loop in TSA plates, supplemented with sodium ampicillin to a final concentration of 10 mgL^-1^, and incubated for 24 h at 28°C, in aerophilic atmosphere [18]. A second tissue fragment was used to characterize the bacterial population. Tissues were weighed (wet weight) and triturated in sterilized micro-tubes, filled with sterile saline phosphate buffer (0.1 M, pH 7.0) at the proportion 1:10 and serially diluted up to 10^−4^. By means of the “Spread-plate” method, 50 μL of each dilution (10^−1^, 10^−2^, 10^−3^ and 10^−4^) and the fresh blood were spread on TSA containing 10 mgL^-1^ of sodium ampicillin. Plates were incubated for 24 h, at 28°C. Then, colony counting was made in an electronic counter. Random samples of the bacterial colonies were confirmed as *A*. *hydrophila* by Polymerase Chain Reaction (PCR).

### Histopathological examination

Tissue samples were fixed in 3.5% neutral buffered formalin for 24 h, and then preserved in alcohol 70%. Then, samples were placed in plastic cassettes and processed by gradual dehydration in 70–100% alcohol, clearing in xylene, and embedding in paraffin wax. Five-micron thick sections were cut using a microtome (Slee, Mainz, Germany), and then stained with hematoxylin and eosin (H&E) [19]. Slides were examined with a light microscope (Olympus BX51 with DP72 digital camera and CellSens Standard 1.5 software package) (Olympus Corporation, Tokyo, Japan).

### Ultrastructural examination

Tissue fragments of spleen, heart, brain, liver and head kidney were fixed in Karnovsky’s solution (2% formaldehyde plus 2.5% glutaraldehyde in phosphate buffer 0.1 M, pH 7.0) for 24 h at 4 ºC, bathed three times in phosphate buffer 0.1 M, pH 7.0, post-fixed in 1% osmium tetroxide in sodium cacodylate buffer (0.1 M, pH 7.2–7.4) for 1 h, and then processed for TEM [20]. The sections were examined using a JEOL-100CXII electron microscope (Jeol, Peabody, MA, USA).

### Statistical analyzes

Data from microbiological analyzes were tested for normality (Kolmogorov-Smirnov, Anderson-Darling, Shapiro-Wilk and Watson) and then submitted to a variance analysis, and Dunn test was applied in order to verify differences within the medians (p < 0.05).

## Results

### Clinical trial and macroscopic alterations

**Fig 1:**
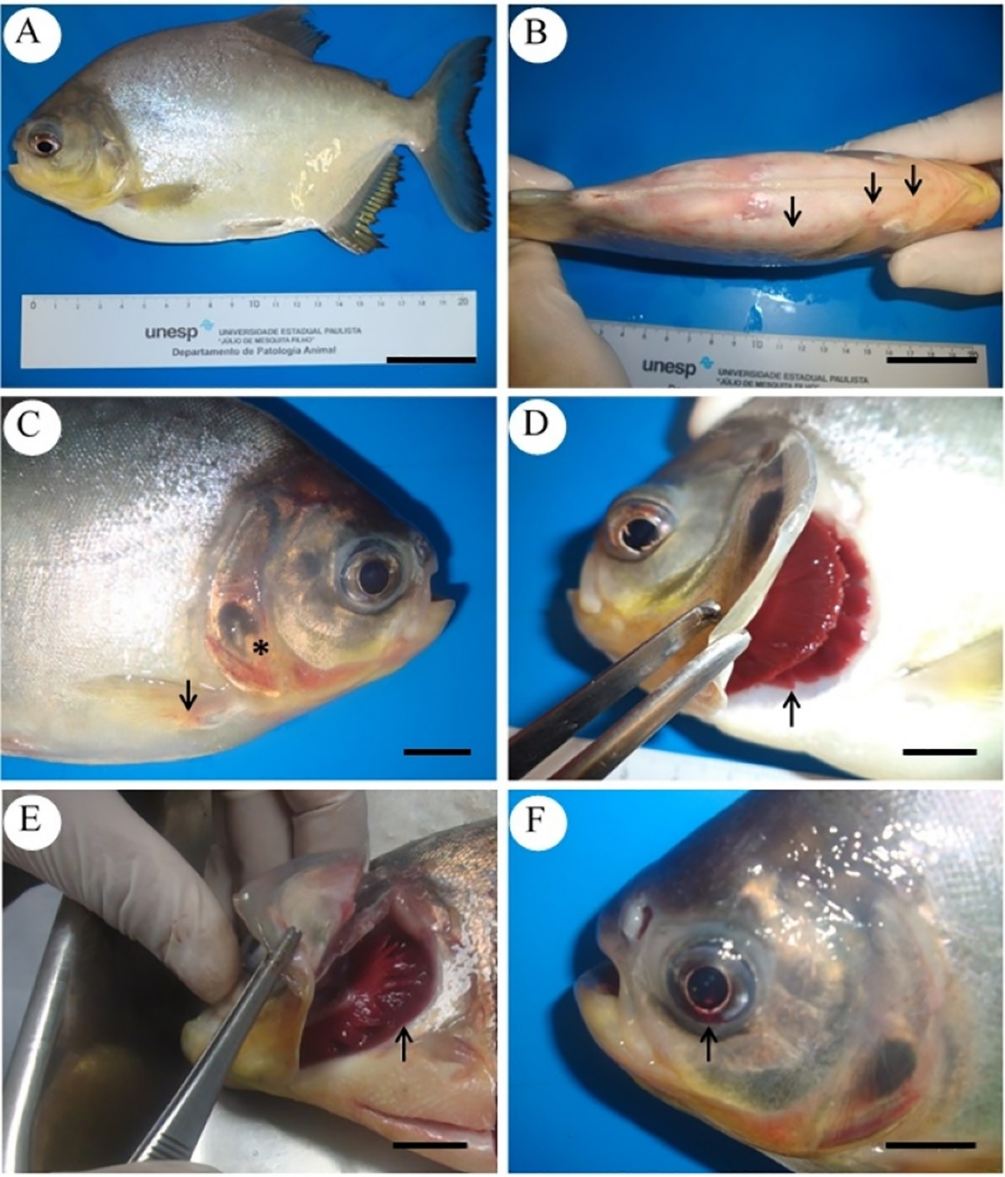
Macroscopic alterations of *Piaractus mesopotamicus* challenged with *Aeromonas hydrophila*. (A) Control (no external changes). (B) Cutaneous haemorrhage (arrows). (C) Hemorrhage at fins (arrow) and operculum (asterisk). (D) Normal gills (arrow). (E) Gill congestion (arrow). (F) Hyphema (arrow). Bars A and B: 4 cm, bars C-F: 1 cm.

**Fig 2:**
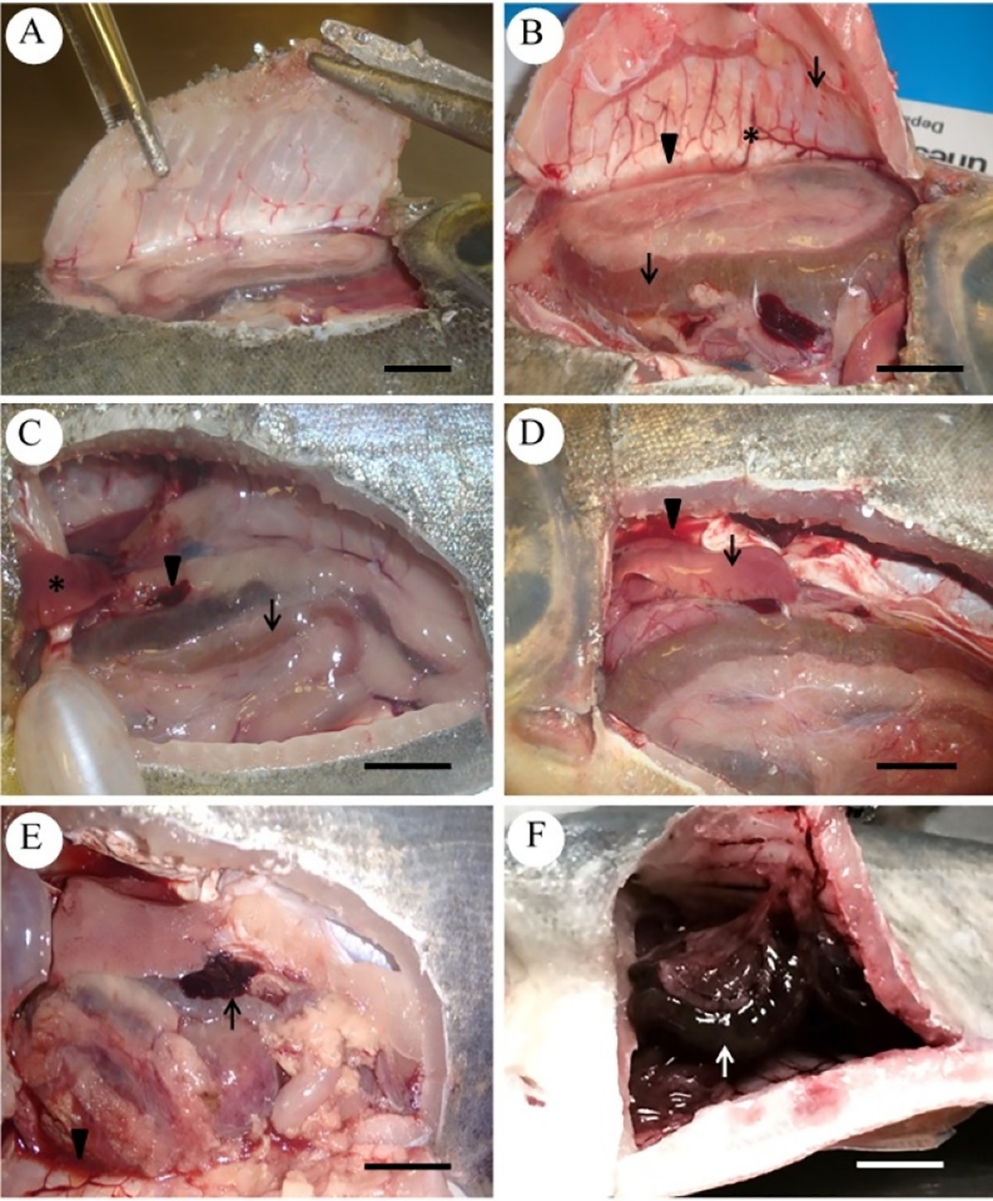
Macroscopic alterations of *Piaractus mesopotamicus* challenged *with Aeromonas hydrophila*. (A) Normal celoma wall. (B) Petechial haemorrhage on the coelomic and visceral walls of the organs (arrows), vessel congestion (asterisk) and presence of serum-sanguineous liquid in coelom (arrowhead). (C) Liver (asterisk), spleen (arrowhead) and bowel (arrow) normal. (D) Hepatic congestion and hepatomegaly (arrow), presence of serum-sanguineous liquid in the coelom (arrowhead). (E) Splenic congestion and splenomegaly (arrow), presence of serum-sanguineous liquid in coelom (arrowhead). (F) Hemorrhagic enteritis (white arrow). Bars: 1 cm.

No behavioral alterations were observed in any of the groups. Macroscopic lesions observed during necropsies were evident only at 6 and 9 hpi, with the last being characterized by a greater severity of lesions. Petechial cutaneous hemorrhages were evident, especially close to the inoculation area (Fig 1B), and in fins and operculum (Fig 1C). Gills congestion (Fig 1E) and the presence of blood in the anterior chamber of the ocular globe (hyphema) were also observed (Fig 1F). The main alterations identified in cavities were petechial hemorrhage at the coelomic and visceral walls of the organs (Fig 2B); congestion of the coelomic wall vessels (Fig 2B); presence of serum-sanguineous liquid in the coelom (Fig 2B–2D and 2E); hepatomegaly and hepatic congestion (Fig 2D); splenomegaly and splenic congestion (Fig 2E); and hemorrhagic enteritis (Fig 2F).

### Microbiological analyzes

**Fig 3:**
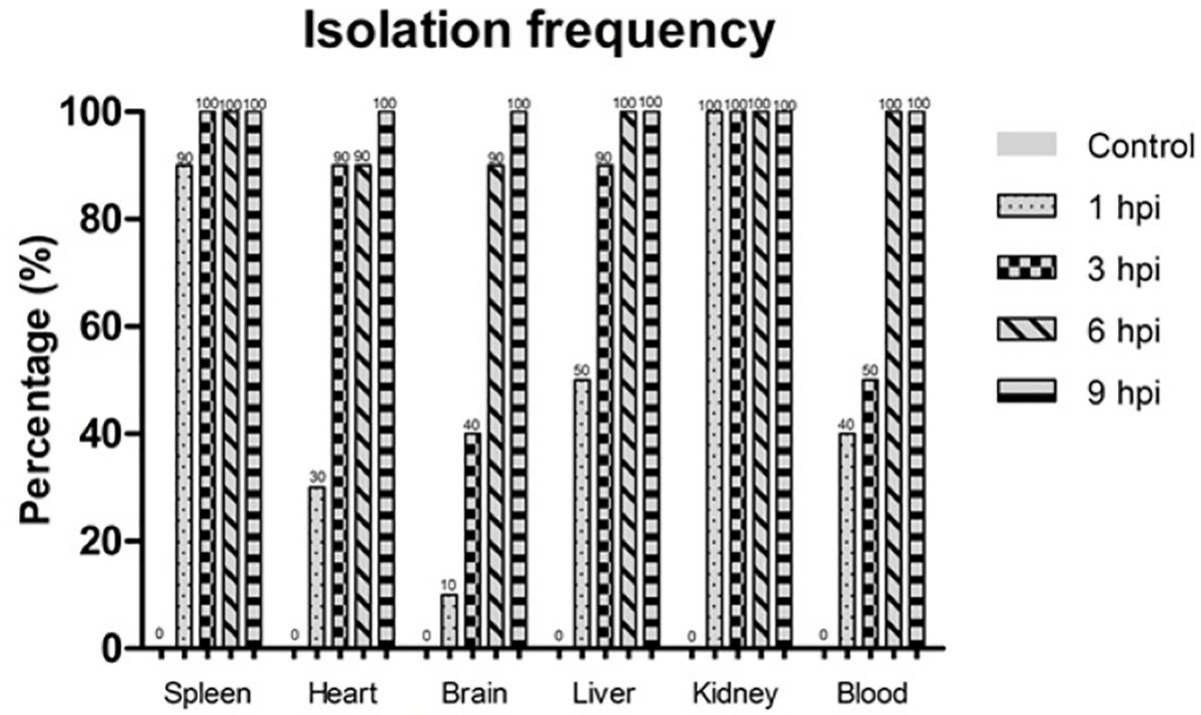
Isolation frequency of *Aeromonas hydrophila* in tissues of infected *Piaractus mesopotamicus*. Vertical columns express the percentages of positive isolation for *A*. *hydrophila* (n = 10) at different times after challenged. hpi = hours post inoculation.

Isolation frequency results were expressed as percentage, characterized by the isolation of pure colonies of *A*. *hydrophila* in different organs and blood after the pre-established experimental times. The data has shown that only at 9 hpi 100% of animals inoculated with the bacteria obtained positive results for *A*. *hydrophila* re-isolation in all analyzed tissues (Fig 3). However, it is noteworthy that at 1 hpi 90% and 100% of animals showed positive results in bacterial recovery from spleen and kidney samples, respectively (Fig 3).

**Fig 4:**
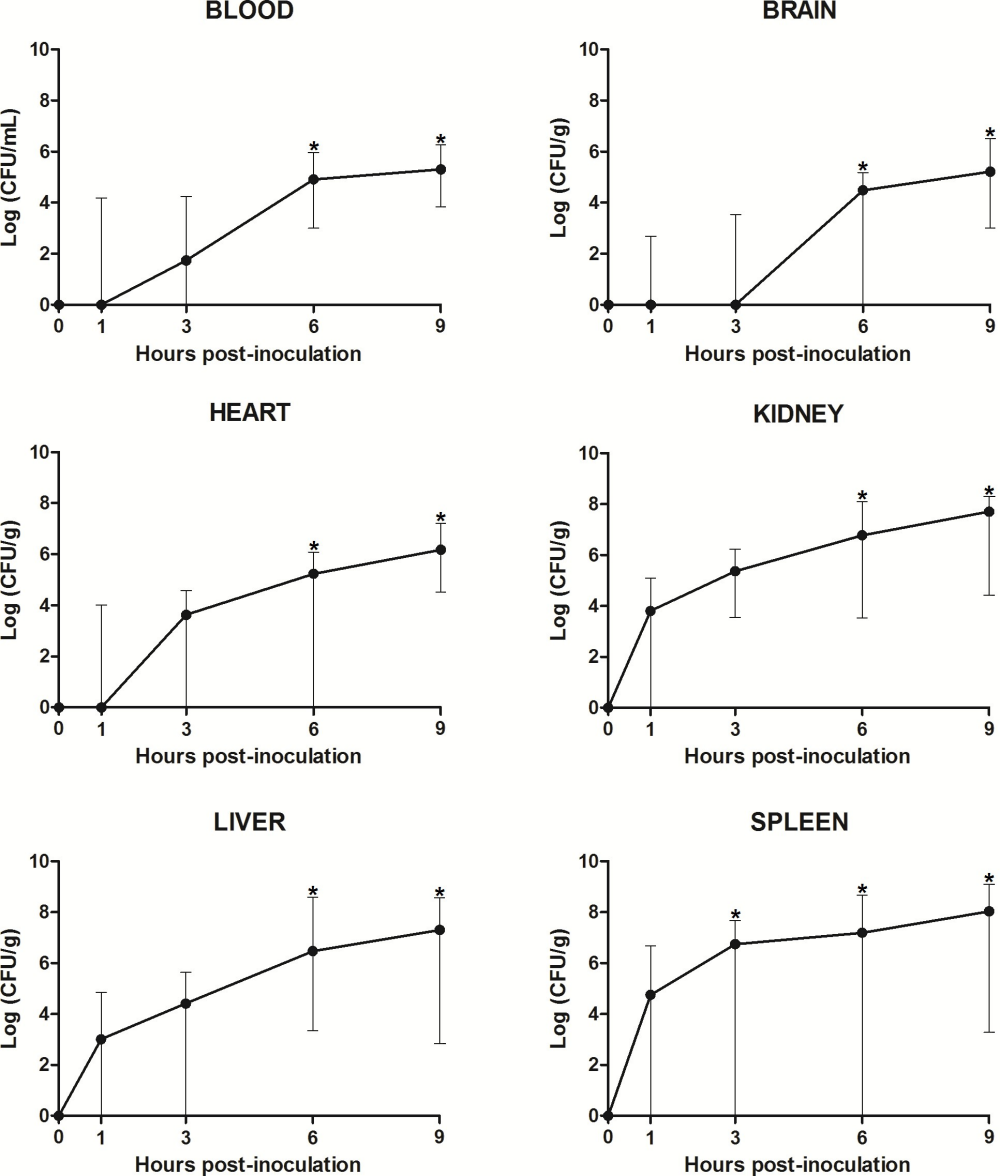
Bacterial population in the organs and blood of *Piaractus mesopotamicus* challenged with *Aeromonas hydrophila*. Bacterial count at 0 (control), 1, 3, 6 and 9 hours post-inoculation (hpi) in each organ and blood is expressed in log of colony forming units per gram (CFU/g). The corresponding amount of bacteria for each group represents the median value and the bars its respective range (n = 10). Asterisks represent significant difference (p < 0.05) between the analyzed times and control group (Dunn's test 5%).

An ascendant bacterial growth was verified in samples of all organs and blood of infected fish throughout time. The greatest amounts of bacteria were verified at 9 and 6 hpi (p < 0.05) compared with the control group (0 hpi), which did not present bacterial growth, in all examined organs and blood (Fig 4). Moreover, at 3 hpi the samples of spleen also displayed significant differences from the control group (Fig 4). It must be emphasized that the tissues with larger bacterial quantities in all experimental times were spleen, kidney and liver.

Macroscopic alterations and microbiological exams showed that the isolated strain of *A*. *hydrophila* from diseased pacus was capable of inducing aeromonosis in healthy fish, which presented characteristic lesions of the disease and presented a positive re-isolation of this bacterial strain in the afflicted tissues of these animals. The strain was confirmed by PCR.

### Histopathological examination

In general, lesions were observed at 6 and 9 hpi in all evaluated organs, being more frequent and intense in the spleen, liver and kidney. The 1 and 3 hpi groups presented no lesions in most of the organs or rare and discrete histopathological alterations, which were characterized by discrete congestion and cellular edema in some organs (e.g., liver, kidney, and gill). Control group was used as a parameter to compare the lesions to the other groups, since it did not show significant histopathological alterations in all organs.

**Fig 5:**
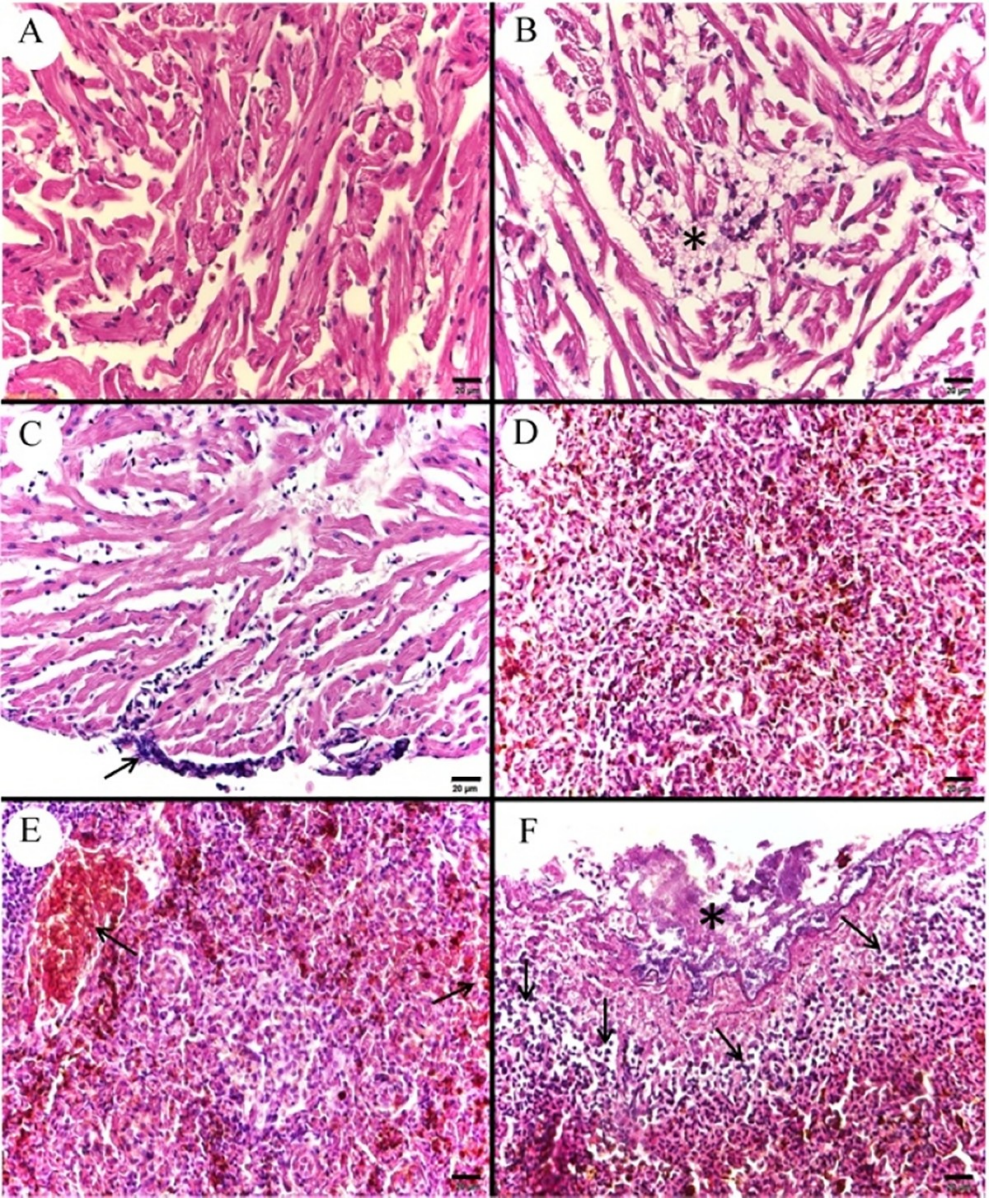
Photomicrograph of control and infected *Piaractus mesopotamicus* heart and spleen. (A) Normal cardiac tissue. (B) Necrosis of ventricular cardiomyocytes and aggregation of inflammatory cells (asterisk). (C) Bacterial colonies adhered to the pericardium and presence of inflammatory cells infiltrates surrounding the area (arrow). (D) Normal splenic tissue. (E) Congestion of spleen vessels (arrows). (F) Bacterial colonies adhered to the splenic capsule (asterisk) and presence of leukocyte infiltrates (arrows). Bars: 20 μm, H&E.

In the heart, bacterial colonies were observed adhered to the pericardium, as well as the presence of leukocyte infiltrate surrounding these areas (Fig 5C); a discrete congestion of pericardium vessels; thrombocyte agglomerates between the ventricle and pericardium; necrosis of ventricle cardiomyocytes and leukocyte infiltrate (Fig 5B). The splenic tissue showed extensive congestion areas (Fig 5E), cytoplasmic vacuolization and cellular edema in the sub-capsular region of the organ. Bacterial colonies were also observed adhered to the splenic capsule, as well as the presence of leukocyte infiltrate (Fig 5F).

**Fig 6:**
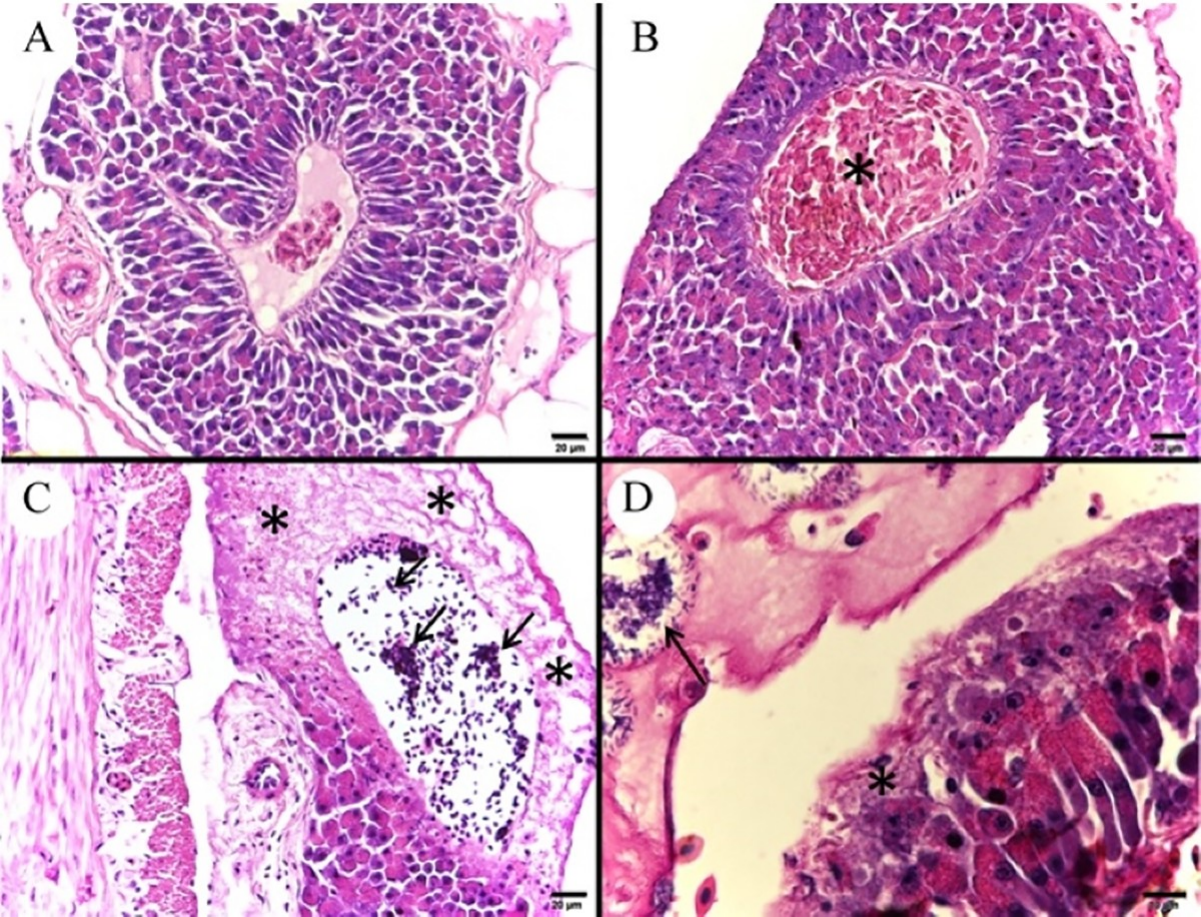
Photomicrograph of control and infected *Piaractus mesopotamicus* exocrine pancreas. (A) Normal exocrine pancreatic tissue. (B) Congestion of large vessels (asterisk). (C) Pancreatic necrosis (asterisks) with dilated vessel and presence of leukocytes inside and surrounding tissue (arrows). (D) Bacterial colonies (arrow) and adjacent pancreatic necrosis (asterisk). Bars A-C: 20 μm and bar D: 10 μm, H&E.

The pancreatic tissue was found in the mesentery of pyloric sacks, in a capsular region of the spleen and liver. We observed extensive areas of cellular death with disorganization of the tissue’s architecture, with the main alterations being the congestion of large vessels (Fig 6B), hemorrhage, extensive areas of necrosis with leukocyte infiltrate (Fig 6C) and the presence of bacterial colonies (Fig 6D).

**Fig 7:**
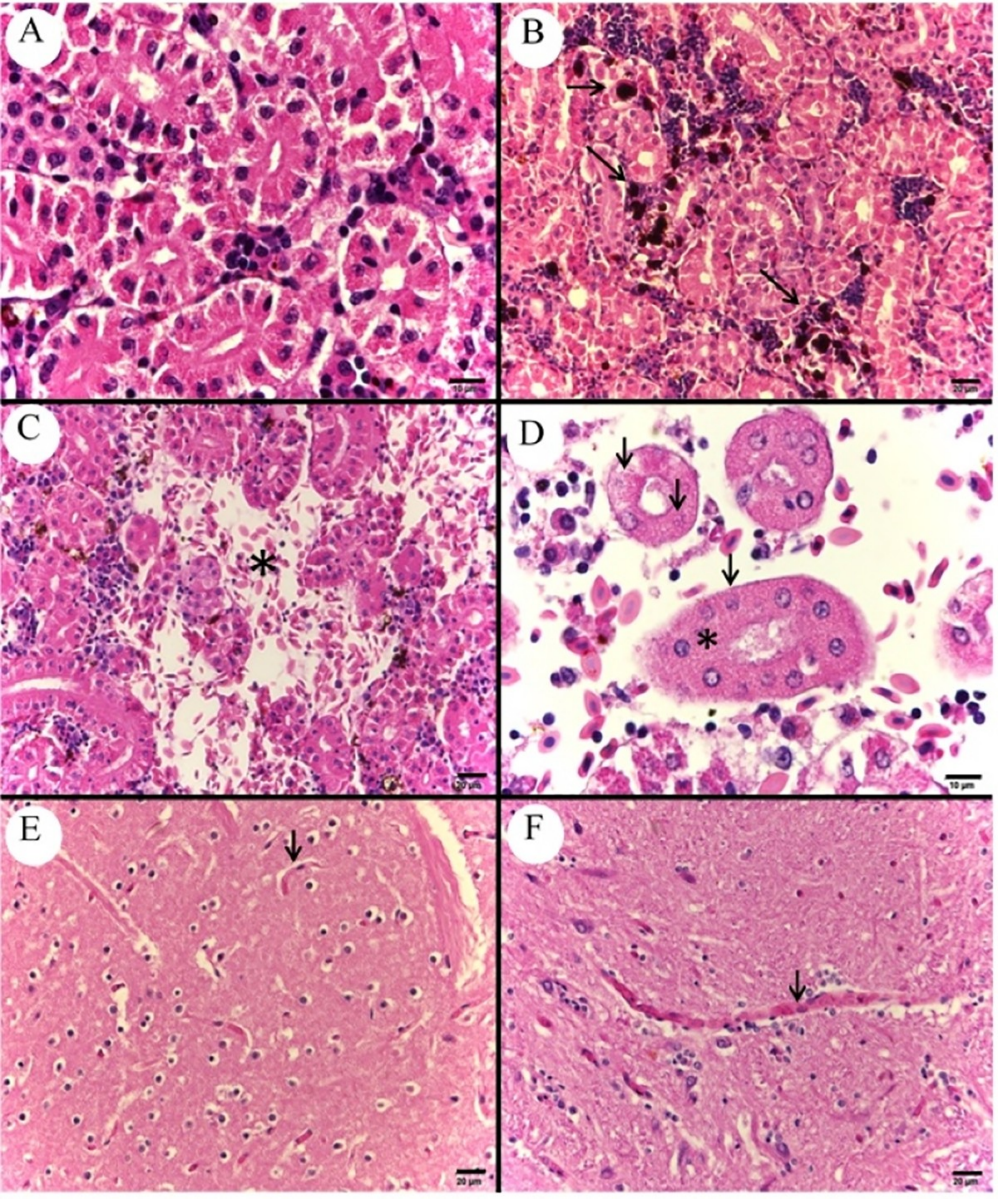
Photomicrograph of control and infected *Piaractus mesopotamicus* kidney and encephalon. (A) Normal organization of renal tubules. (B) Abundant melanomacrophages accumulation between renal tubules (arrows). (C) Interstitial hemorrhage in renal tissue (asterisk). (D) Necrosis with karyolysis of tubular cell’s nucleus (arrows) and loss of cytoplasmic delimitation between renal tubule epithelial cells (asterisk). (E) Capillary of normal nervous tissue without congestion (arrow). (F) Discrete capillary congestion in nervous tissue (arrow). Bars A and D: 10 μm, bars B, C, E and F: 20 μm, H&E.

In the kidney, larger quantities of melanomacrophages were found (Fig 7B), as well as hemorrhage (Fig 7C), necrosis with karyolysis and loss of the cytoplasmic delimitation of cells of the renal tubules (Fig 7D), congestion of large vessels and presence of bacterial colonies surrounded by leukocytes. Discrete capillary congestions were also verified in the encephalon (Fig 7F).

**Fig 8:**
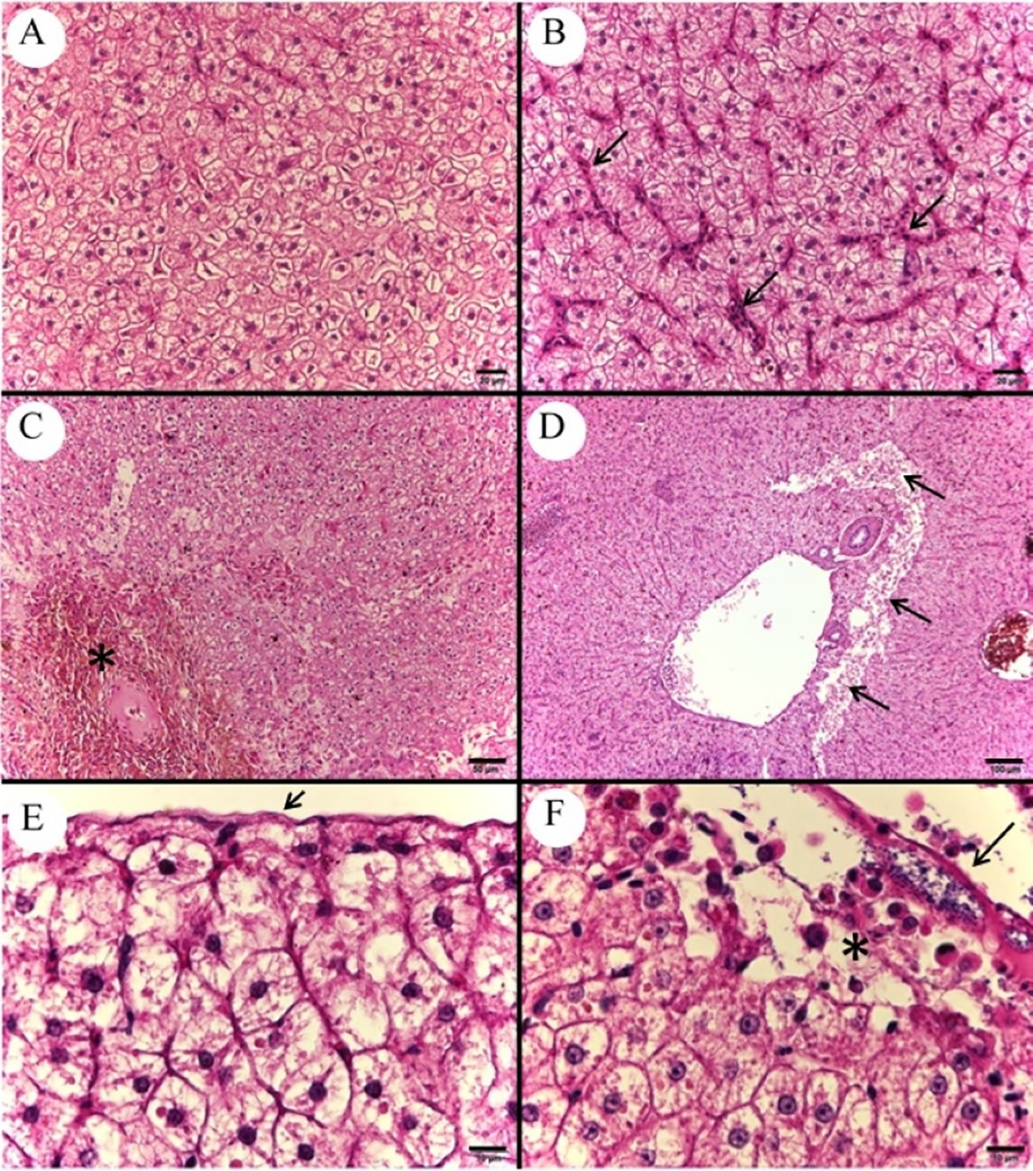
Photomicrograph of control and infected *Piaractus mesopotamicus* liver. (A) Control liver tissue. (B) Congestion of hepatic sinusoids (arrows). (C) Hepatic hemorrhage (asterisk). (D) Hepatic necrosis and disorganization of hepatic tissue architecture in perivascular region (arrows). (E) Normal hepatic capsule (arrow). (F) Bacterial colonies adhered to the hepatic capsule (arrow) with leukocyte infiltrate and hepatocyte necrosis (asterisk). Bars A and B: 20 ​​μm, bar C: 50 μm, bar D: 100 μm and bars E and F: 10 μm, H&E.

Sinusoidal capillaries and large vessels congestion was verified in the liver (Fig 8B), as well as hemorrhage (Fig 8C), cellular edema in hepatocytes; disorganization of the hepatic tissue’s architecture, especially below the capsule and in perivascular regions (Fig 8D), presence of bacterial colonies adhered to the hepatic capsule with leukocyte infiltrates and hepatocyte necrosis (Fig 8F).

**Fig 9:**
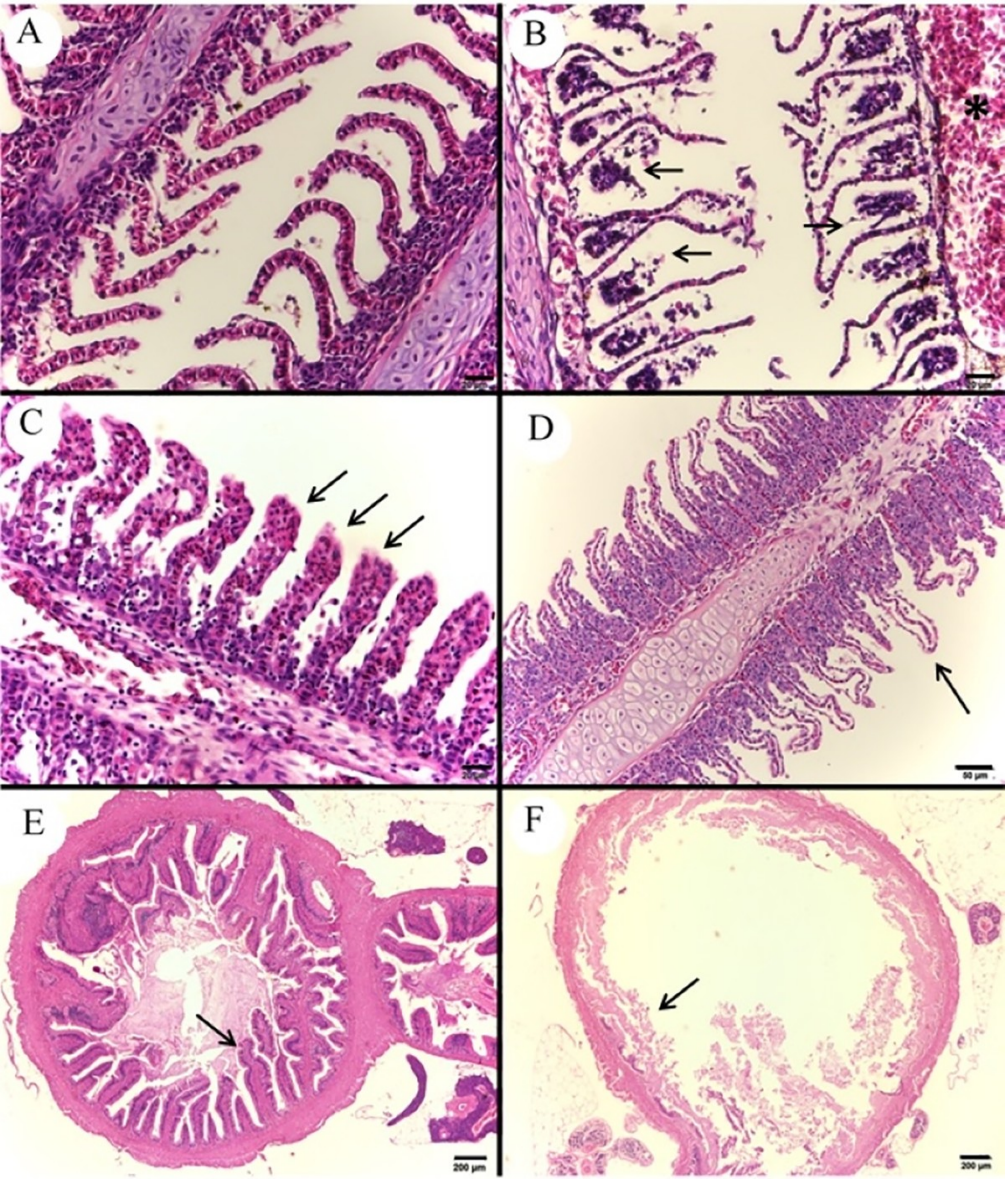
Photomicrograph of control and infected *Piaractus mesopotamicus* gill and intestine. (A) Gill filaments and normal secondary lamellae tissue. (B) Detachment of epithelial cells from the lamella base that are thinned (arrows) and congestion of a large vessel (asterisk). (C) Congestion of secondary lamellae (arrows). (D) Secondary lamella edema (arrow). (E) Normal villi and intestinal mucosa (arrow). (F) Necrosis of the villi and intestinal mucosa (arrow). Bars A-C: 20 μm, bar D: 50 μm and bars E and F: 200 μm, H&E.

Infected fish presented congestion of large vessels of gill filaments and extensive areas of epithelial cells detachment at the base of interlamellar spaces (Fig 9B). Other findings in gills were congestion (Fig 9C), interlayer hyperplasia and subendothelial edema (Fig 9D) of secondary lamellae. The intestines presented villi and intestinal mucous necrosis (Fig 9F) and presence of bacterial colonies adhered to both serum and muscular layers.

### Ultrastructural examination

**Fig 10:**
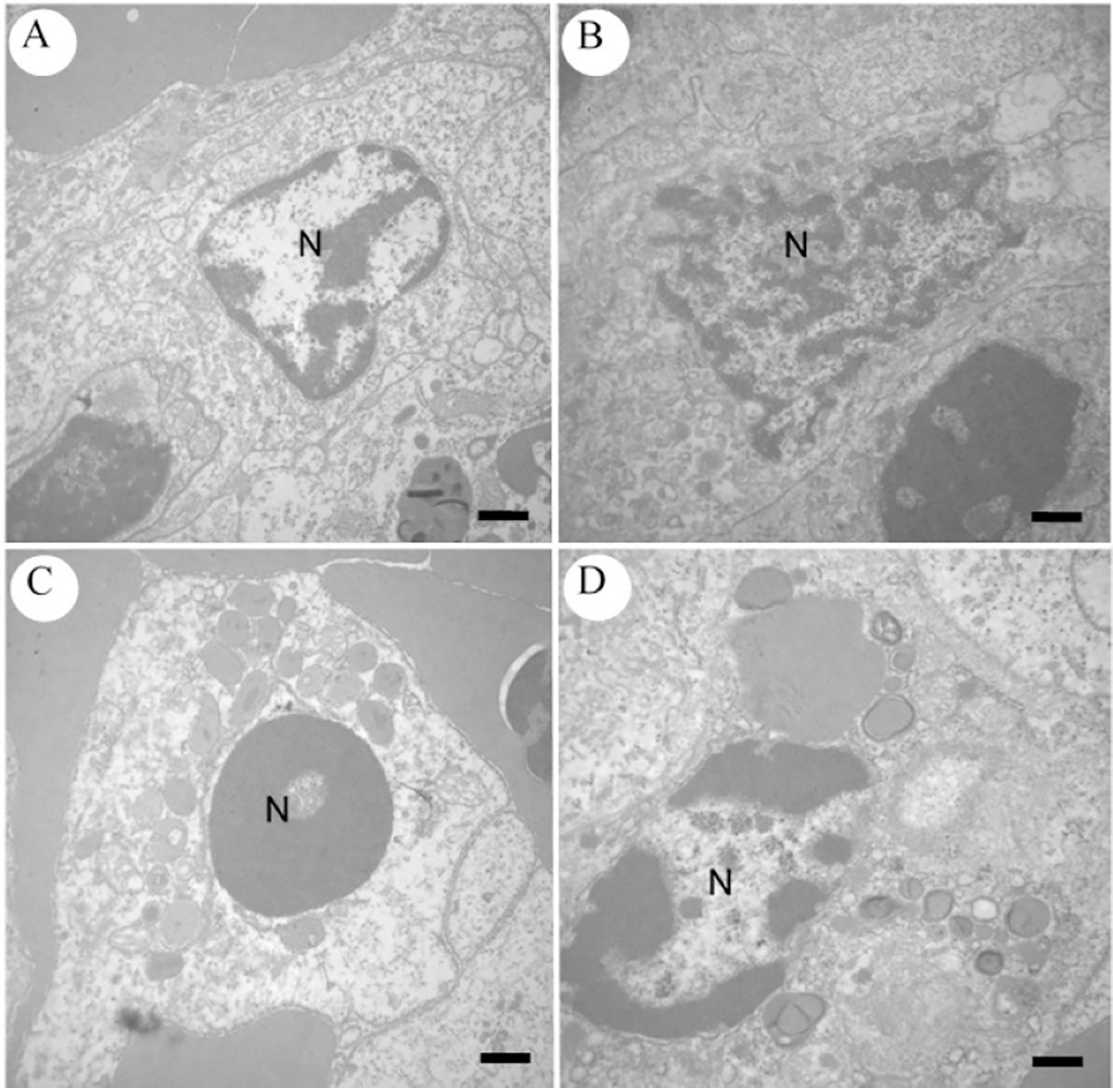
Transmission electron microscopy images of control and infected spleen cells of *Piaractus mesopotamicus*. (A) Normal nucleus (N) in a lymphocyte. (B) Nuclei in karyolysis (N) in a lymphocyte. (C) Pynotic nucleus (N) in a lymphocyte. (D) Nucleus in karyorrhexis (N) in a lymphocyte. Bars: 1 μm.

**Fig 11:**
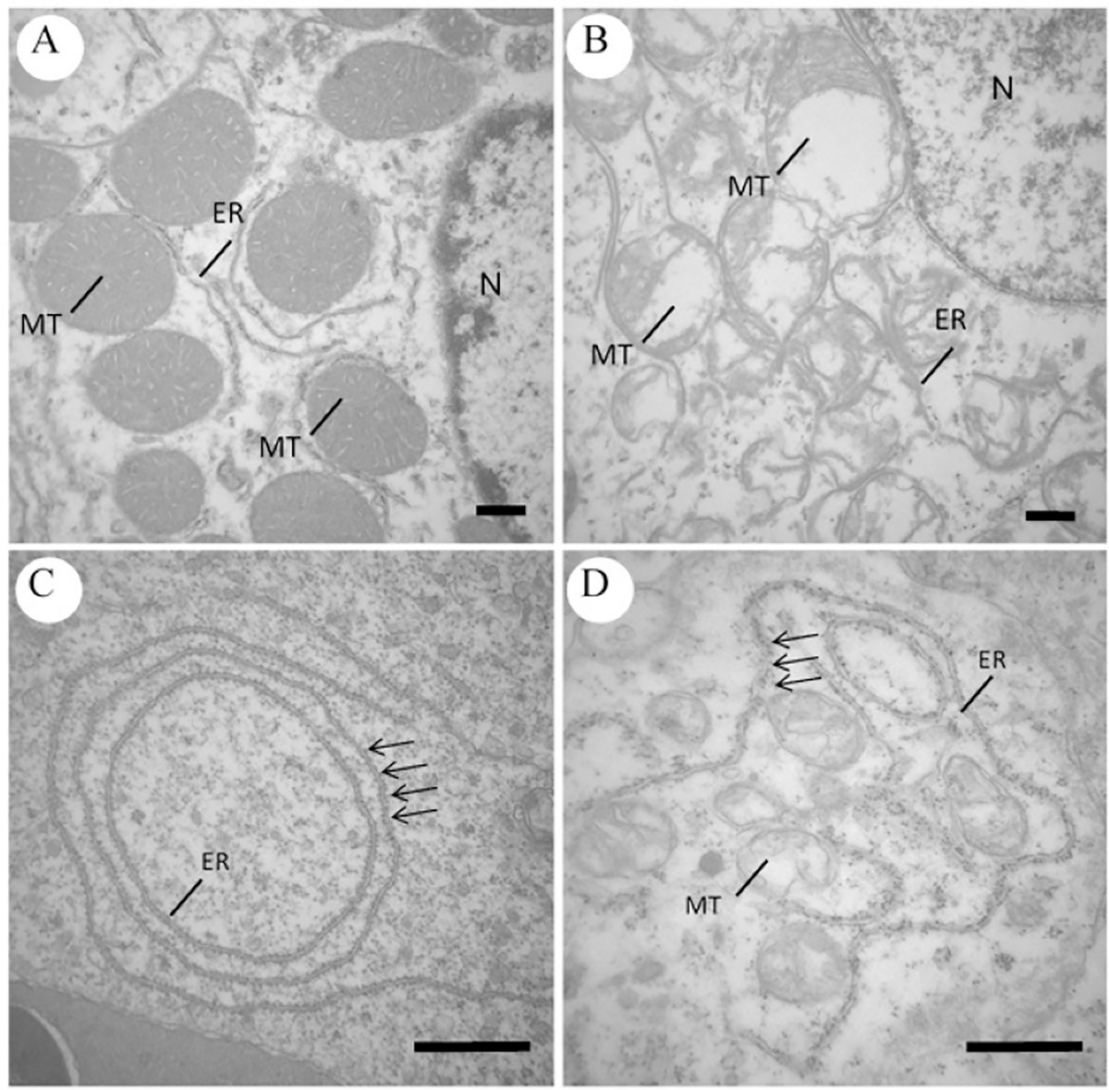
Transmission electron microscopy images of control and infected kidney and heart cells of *Piaractus mesopotamicus*. (A) Normal renal tubule cell showing mitochondria (MT), endoplasmic reticulum (ER) and nucleus (N). (B) Infected renal tubule cell with loss of membrane integrity and dissolution of mitochondria (MT) and endoplasmic reticulum (ER). (C) Normal cardiomyocytes showing endoplasmic reticulum (ER) with adhered ribosomes (arrows). (D) Dilatation and detachment of ribosomes (arrows) from endoplasmic reticulum (ER) of infected cardiomyocytes. Bars: 1 μm.

**Fig 12:**
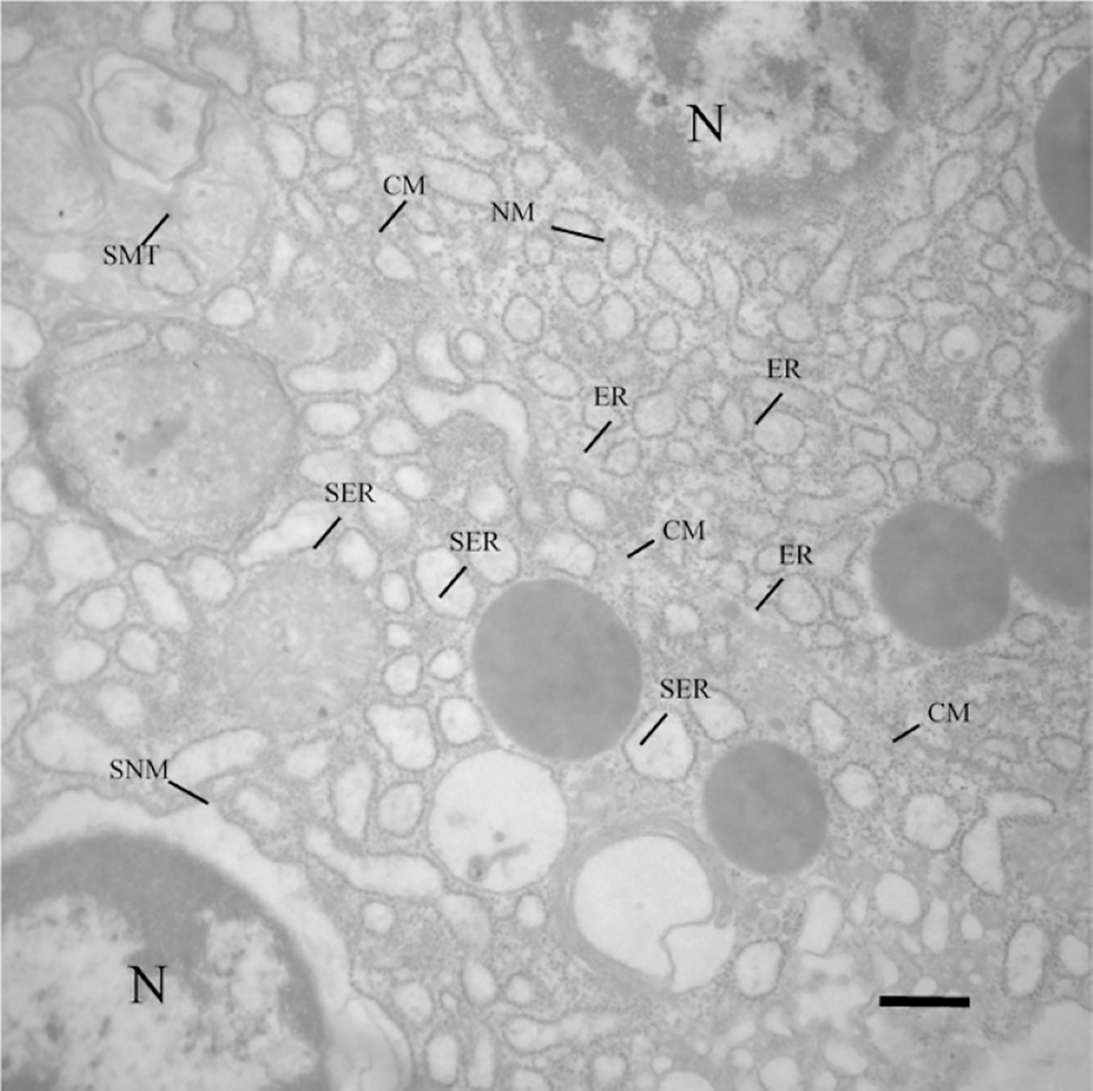
Transmission electron microscopy image of infected kidney cells of *Piaractus mesopotamicus*. A renal tubule cell is showed in the upper right portion of the image, with normal nucleus (N), nuclear membrane (NM) and endoplasmic reticulum (ER). Separated by the cytoplasmic membrane (CM), another renal tubule cell located in the lower left portion of the image, shows endoplasmic reticulum edema (SER), nuclear membrane (SNM) and mitochondria (SMT). Bar: 1 μm.

**Fig 13:**
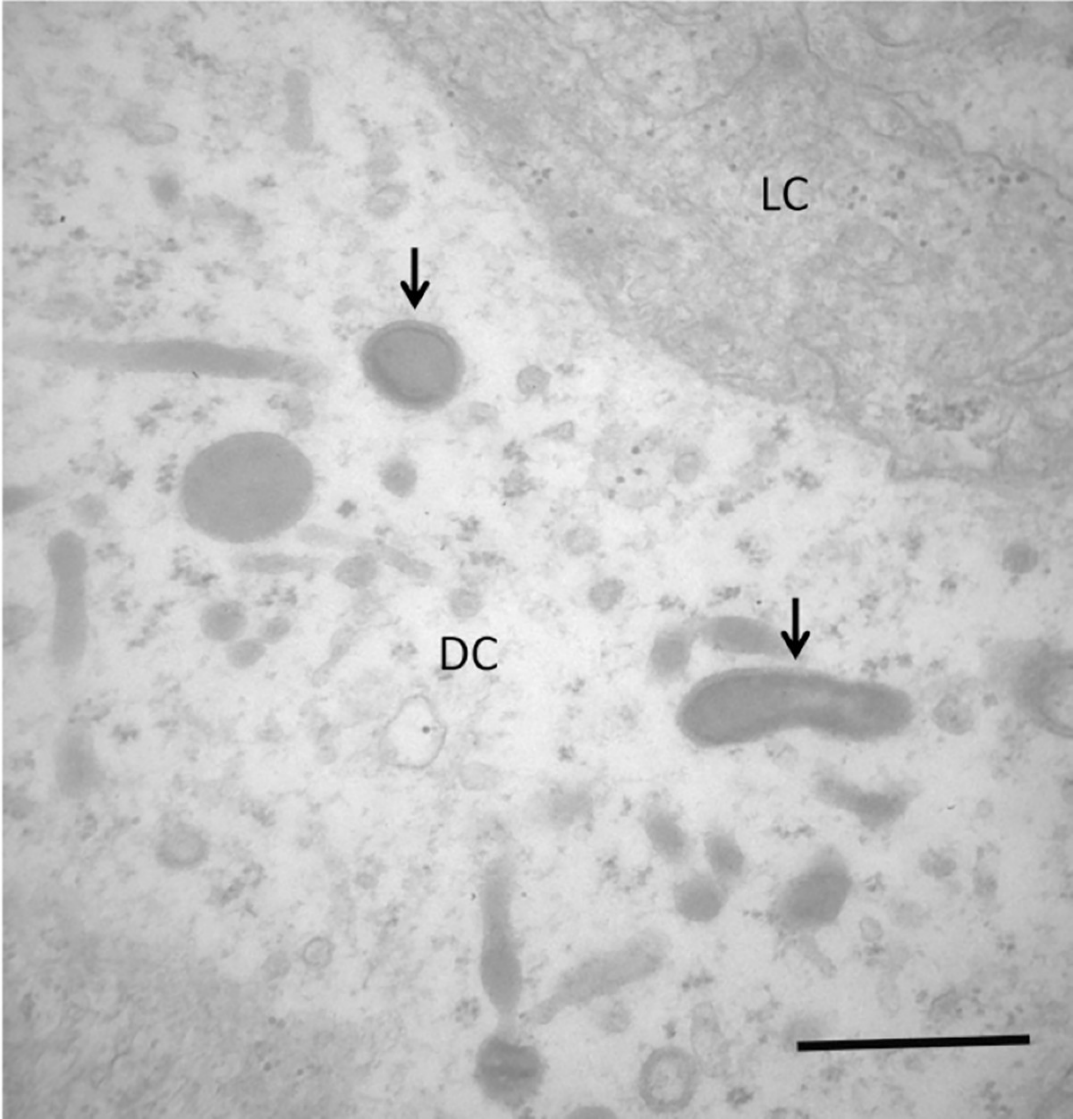
Transmission electron microscopy image of infected splenic hemopoietic precursor cells of *Piaractus mesopotamicus*. Bacteria presence (arrows) inside a compromised cytoplasm of a dead cell or in the process of death (DC). A normal cytoplasm of a living cell (LC) is shown for comparison. Bar: 1 μm.

**Fig 14:**
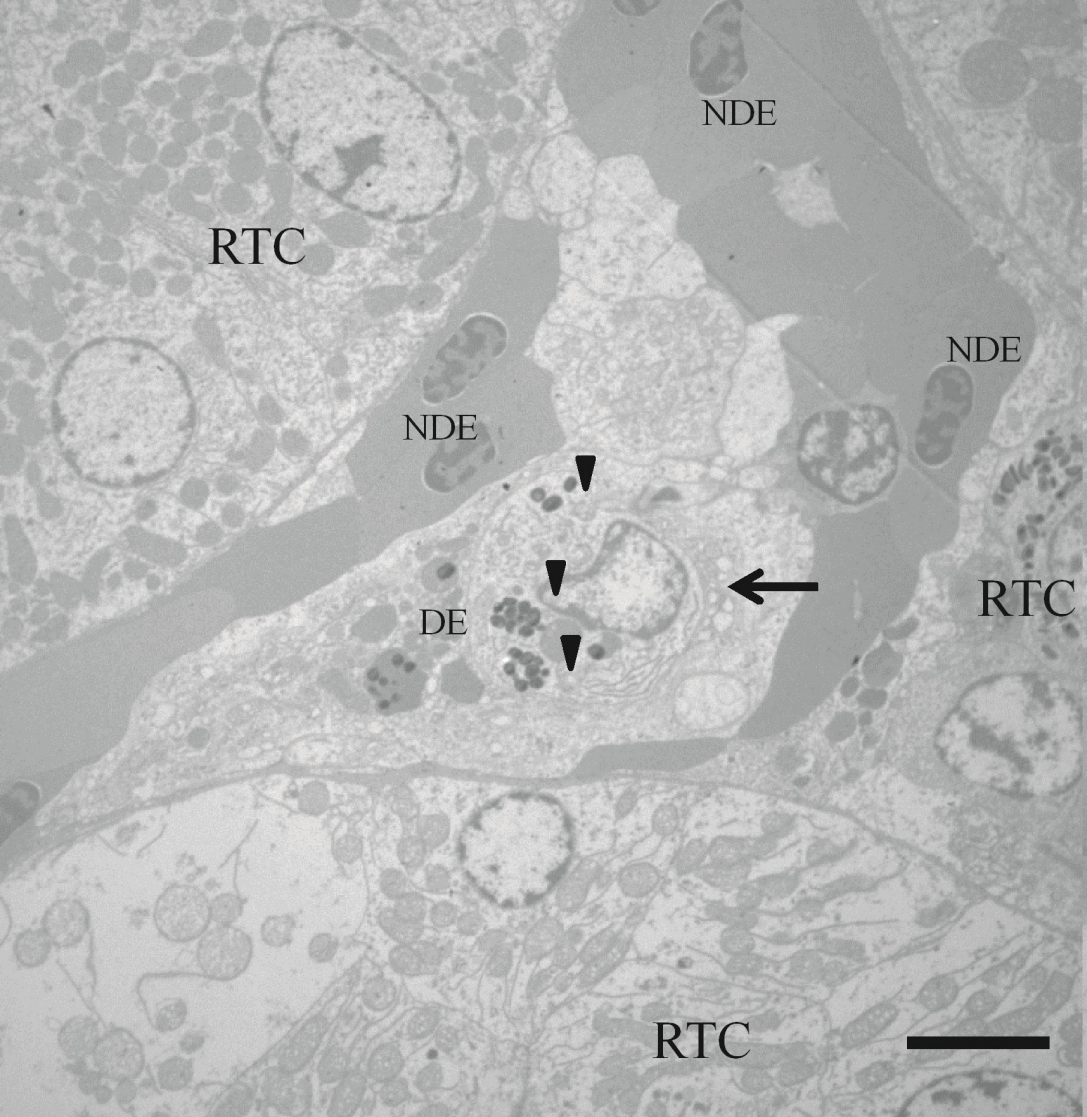
Transmission electron microscopy image of infected kidney cells of *Piaractus mesopotamicus*. Phagocytosis process of the products of erythrocyte degradation, note the presence of a melanomacrophage (arrow) probably engulfing the products of a degraded erythrocyte (DE) and forming siderosomes (arrowhead) inside its cytoplasm, it also can be seen non-degraded erythrocytes (NDE) and renal tubule cells (RTC). Bar: 1 μm.

Extensive areas of cellular death were identified in all examined tissues, especially at 6 and 9 hpi, in which the findings were more severe, in comparison to groups 1 hpi, 3 hpi and control. The most common ultrastructural alterations observed at the present study were karyolysis or chromatolysis (Fig 10B); condensation of chromatin or pyknosis (Fig 10C); fragmentation of chromatin or karyorrhexis (Fig 10D); cytoplasmic and organelle membranes integrity loss and organelle dissolution (Fig 11B); dilation and detachment of ribosomes from the endoplasmic reticulum (ER) (Fig 11D); edema of ER, nuclear membrane and mitochondria (Fig 12); presence of erythrocytes out of vessels, indicating hemorrhage and presence of bacteria inside dead and disorganized cells (Fig 13); and phagocytosis of the products of erythrocyte degradation by melanomacrophages (Fig 14).

## Discussion

This study showed the kinetics of histopathological lesions in the acute stage of experimental septicemic aeromonosis in pacus, revealing that the appearance of macro and microscopic lesions initiates at 6 hours post-inoculation (hpi), with more severe results at 9 hpi. The development and severity of lesions are related to bacteria pathogenicity, seen that *Aeromonas* are pathogens that have several virulence factors, thus the infection is complex and multifactorial [21–23].

Altered behavioral manifestations were not shown by fish throughout the clinical observation period up to and including 9 hpi, which may be related to the absence of severe injuries in its nervous system; neurological disturbances such as erratic swimming are one of the main behavioral alterations in fish affected by aeromonosis [24,25]. Another hypothesis that may explain the absence of behavioral alterations is related to the incubation period and neurological signs in this disease, in other words only 9 hpi may not be enough for such manifestations to appear in pacus. It was showed that in the acute phase of *A*. *salmonicida* infection in zebrafish, the signs related to swimming disturbances occurred only 12 hours after the experimental infection [26].

Concerning macroscopic examinations, inoculated fish presented petechiae hemorrhage in the body, fins and operculum surfaces, gills congestion and ocular hemorrhage. Similarly, studies of *Aeromonas* spp. infection in cyprinids, demonstrated external macroscopic findings, such as skin darkening and hemorrhage in the body surface, fins, gills and eyes [27,28].

The findings regarding the necropsy performed using fish evidenced hemorrhages in serous and organs, a discrete accumulation of serosanguinolent liquid in the cavity, congestion and a slight increase of the organs and superficial vessels volume, corroborating the results described in the literature for others species of fish affected by aeromonosis [29]. Similarly, Fichi et al. [28] observed the presence of hemorrhage in internal organs (heart, kidney, swim bladder and ovary) of crucian carps (*Carassius carassius*) infected by *A*. *sobria*.

Macroscopic hemorrhagic alterations possibly occurred due to the acute inflammatory response to the infection. Leukocytes release several vasoactive substances that cause vasodilation and increased vascular permeability, leading to hemorrhages [30–32].

Microbiological examination revealed that, even in low percentages, bacteria were detected at the first hour post-inoculation (1 hpi), reaching 100% of positive isolations at 9 hpi in all organs and blood of infected fish. This rapid dissemination of *A*. *hydrophila* through the tissues supposedly occurred as a function of the motile capacity of these bacteria, which moves with the aid of flagella.

One of the most important virulence factors of *Aeromonas* spp. in the initial colonization of tissues relates to the presence of polar flagella, which allows the organism to move in liquid environments, and lateral flagella, aiding in viscous surfaces, giving a greater invasive capacity for these bacteria [33]. In studies of *Tenacibaculum maritimum* infection, a motile microorganism that does not have flagella and moves by sliding, Faílde et al. [34] detected its presence in internal organs of infected fish (*Psetta maxima* L.) only six hours after experimental inoculation. By comparing these data with our findings, it can be inferred that the presence of flagella may have conferred a greater precocity in the initial colonization of pacus tissues, seen that *A*. *hydrophila* was already found in the first hour after infection.

The upward curve of bacterial quantification in organs and blood, emphasizing the greatest values for spleen, liver and kidney in all experimental times. This fact may indicate that these organs are most susceptible to infection by *A*. *hydrophila* in pacus, thus facilitating the diagnosis of this pathology, even in early cases through the preventive diagnosis of outbreaks. In studies of acute experimental streptococcosis, Abdullah et al. [35] isolated bacterial colonies from the encephalon, eyes and kidney, four hours after coelomatic inoculation of *Streptococcus agalactiae* in red tilapia (*Oreochromis* spp.), determining the encephalon as the target-organ of this microorganism in this fish.

Bacterial virulence factors are related to the capacity of invasion, replication and evasion of the host immune system, causing lesions during pathogenesis [36]. Farto et al. [25] studied the capacity of invasion and distribution of three strains of *A*. *salmonicida* subspecies *salmonicida*, two avirulent and one considered virulent, in organs of experimentally infected preached fish (*Psetta maxima* L.). The results indicated that the virulent strain was isolated in internal organs, especially liver and kidney, in the first 12 hpi, which remained present in all experimental times until death, after seven days. Conversely, avirulent strains were rarely isolated from internal organs, being eliminated four days after the challenge.

Histopathological findings showed that *A*. *hydrophila*, when directly inoculated in the coelom of pacus, causes lesions in multiple organs. These lesions were observed at 6 hpi and were more severe at 9 hpi. As *Aeromonas* spp. possesses the capacity of adhering to tissue cells, the illness severity depends on the types of virulence factors involved and the immune *status* of the host, which leads to several degrees of injury on tissues [37–39].

Histopathological lesions occurred likely due to the liberation of bacterial toxins and other virulence factors inherent to aeromonosis. This bacterium produces endotoxins and exotoxins, such as hemolysin and aerolysin, which cause the rupture of cellular membranes [40,41], enterotoxins [42], dermonecrotic factors, proteases, phospholipases and DNAses that causes tissue damage and facilitate bacterial invasion and multiplication in the hosts’ cells [40,42].

The present results showed that, after the bacteria inoculation in the coelom, probably, there was colonization of serosa or capsular compartment of the organs in this cavity (e.g., liver, spleen, heart, kidney, intestine, and pancreas). Following that colonization, bacteria crossed the epithelial barriers and leaded to the infection and, consequently, inducing damage to the cells inside these organs. Concomitantly, some bacteria crossed mesothelium of peritoneum vessels and reached the blood circulation and colonized even distant organs (e.g., brain and gills). Farto et al. [25] observed the same colonization mechanisms of turbot tissues infected with *A*. *salmonicida* subsp. *salmonicida*.

No available reports of histopathological findings in the acute phase of aeromonosis were found until this time, however studies on advanced stages of septicemic diseases caused by pathogenic bacteria in fish showed similar histopathological alterations to the ones found in this study. Aguado-Urda et al. [43] observed necrosis in the renal tubular epithelium and hemorrhage in compact layers of the heart after experimental infection with *Lactococcus garvieae* in zebrafish. Chong et al. [44] found, in Australian eels inoculated with *Erysipelothrix rhusiopathiae*, moderate brain and hepatic vascular congestion. Carraschi et al. [45] reported sub-epithelial edema in secondary gill lamellae in pacus infected with *A*. *hydrophila*, and Avci et al. [46] found the same histopathological findings in rainbow trouts infected with *Lactococcus garvieae*.

Bacteria that causes sepsis are frequently observed in injured areas or associated to inflammatory processes in teleosts previously challenged or naturally infected [43,44,47], corroborating with the presence of the observed bacterial colonies in the lesions of this study. This occurs due to the structural components of *Aeromonas* spp., such as flagella, fimbria, membrane proteins and lipopolysaccharides (LPS), which allows the adherence of bacteria to the fish tissues [43].

Similar to our histopathological findings in kidney and liver, other studies of bacterial septicemia in fish reported that hybrid catfish (*Clarias macrocephalus* x *Clarias gariepinus*) infected with *Edwardsiella ictaluri* presented lymphocyte infiltrate in the renal glomeruli [48] and cyprinids (*Puntius sarana*) challenged with *A*. *hydrophila* presented increased number of melanomacrophages and congestion of central vessels in the liver [27].

The increased quantities of melanomacrophages in pacus infected with *A*. *hydrophila* is possibly related to the lysis of red blood cells, caused by bacterial toxins such as hemolysin and aerolysin, seen that these cells are responsible for the phagocytosis of the products of erythrocyte degradation, such as hemosiderin [49]. This is another indicative of the action of toxins in organs of pacu, throughout the acute stage of aeromonosis, since the increase of melanomacrophages relates to the protective response of tissues against damages caused by free radicals [27].

In this study, the observed lesions in transmission electron microscopy reinforce the histopathological observations, cellular death was verified, and organelle degeneration and a strict association between lesions and the presence of bacteria in infected organs. Likewise, eels (*Anguilla anguilla*) infected with *Vibrio vulnificus* manifested the presence of the bacteria in a close association with necrotic cells, which presented alterations in the cell membrane and organelle damage [20].

In a study concerning the resistance of *Streptococcus iniae* inside macrophage phagosomes of gilthead seabream (*Sparus aurata*) and red porgy (*Pagrus pagrus*), the authors demonstrated by means of ultrastructural analyzes, that these cells may be important sources of bacteria dissemination in the nervous system, since bacteria may cross the blood-brain barrier without any resistance [50].

The organs that presented more severe microscopic lesions were liver, spleen and kidney. These organs also presented the greatest bacterial quantifications, indicating that these are the target organs of the acute stage of septicemic aeromonosis in pacu. Monaghan et al. [51] determined through *in situ* hybridization, that both gills and intestines are target organs of the acute infection by herpesvirus in carps (*Cyprinus carpio*) after challenged by immersion. According to these authors, these organs were relevant as a gateway in the initial stage of the illness, reinforcing the importance of the determination of target organs in the understanding of infectious diseases.

The findings showed that this bacterium spreads in the first hpi through the pacus organs. Morphological and microbiological findings showed necrosis, degenerative processes, vascular alterations and an association between bacteria and lesions, especially at 6 and 9 hpi, demonstrating the action of the virulence factors of *A*. *hydrophila* in the pathophysiology of the acute stage of septicemic aeromonosis in pacu organs. Spleen, liver and kidneys were the most affected organs and are suggested as the target organs of the infection, which may assist the diagnosis of acute cases of this pathology in pacus. These findings help to improve the knowledge about the infection process model and may orientate and assist the diagnosis of acute cases of this pathology in pacus.
